# Systemic osteoprotective effects of *Epimedii Folium* and *Ligustri Lucidi Fructus* in senile osteoporosis rats by promoting the osteoblastogenesis and osteoclastogenesis based on MLP-ANN model

**DOI:** 10.1186/s13020-020-00368-0

**Published:** 2020-08-20

**Authors:** Xiu-Feng Tang, Zi-Tong Ma, Ying-Ying Gao, Han Wang, Xiao-Xi Li, Ping Yu, Ren-Hui Liu

**Affiliations:** 1 Shandong Tumor Hospital and Institute, Jinan, Shandong China; 2 School of Traditional Chinese Medicine, Capital Medical University, No.10 Xitoutiao, Youanmenwai, Fengtai District, Beijing, 100069 China

**Keywords:** Senile osteoporosis, Tonifying-*shen* (kidney) prescription, Bone remodeling

## Abstract

**Background:**

Senile osteoporosis (SOP), which is caused by unbalanced bone remodeling, leads to significant economic and societal burdens globally. The combination of *Epimedii Folium* (EF) and *Ligustri Lucidi Fructus* (LLF) serves as a commonly-used prescription for SOP in Traditional Chinese Medicine (TCM). This study aimed to evaluate the osteoprotective effects of EF and LLF in combination on SOP rats based on the constructed multilayer perception (MLP)-artificial neural network (ANN) model.

**Methods:**

15 month old male Sprague–Dawley rats were administrated with EF, LLF or the combination of EF and LLF (EF&LLF) for 2 months, while 17 month old rats were used as the aging control group. All the rats were anesthetized with 25% ethyl carbamate, then their serum liver and bone tissues were taken. We detected bone mass, bone mineral density (BMD), biomechanics and the microstructure of bone trabecula by micro-CT and H&E staining to evaluate the degree of osteoporosis. Blood lipids and serum alanine aminotransferase (ALT), aspartate aminotransferase (AST) and γ-glutamyl transferase (GGT) and liver pathology were use to assess the side effects of drugs. Levels of alkaline phosphatase (ALP) and Tartrate-resistant acid phosphatase (TRACP) and the ratio of ALP to TRACP both in serum and bone were measured for the evaluation of bone turnover rate. The bone mRNA and protein expression of osteoprotegerin (OPG), nuclear factor-kappa B ligand (RANKL), macrophage colony-stimulating factor (M-CSF), d2 isoform of vacuolar (H^+^) ATPase (ATP6V0d2), insulin-like growth factor (IGF-1), bone morphogenetic protein-2 (BMP2), M-CSF, Wnt5a, transforming growth factor-β1 (TGF-β1) were detected for evaluating bone metabolism.

**Results:**

The results showed that EF&LLF improved bone mass and bone quality by preventing bone loss, increasing maximal load as well as protecting the micro-structural retrogressive change of trabecular bone in SOP rats; ameliorated the steatosis in the liver and decreased blood lipids and serum ALT, AST and GGT; enhanced bone remodeling by stimulating the expression of ALP and TRACP. At the molecular levels, EF&LLF stimulated the osteoclastogenesis by upregulating the protein and mRNA expression of OPG, RANKL, M-CSF and ATP6V0d2; meanwhile, EF&LLF stimulated osteoblastogenesis by enhancing the expression of TGF-β1, BMP2, Wnt5a and IGF-1. According to our established MLP model, EF&LLF has a better effect on osteoclastogenesis or steoblastogenesis in SOP rats than EF or LLF.

**Conclusions:**

These findings demonstrate that the systemic bone protective effects of EF&LLF by promoting bone remodeling in aging rats might be a substitute medicine for the treatment of SOP.

## Background

Osteoporosis (OP) is a systemic disease characterized by low bone mass and disruption of bone microarchitecture, resulting in increased bone fragility and prone to fracture [1]. Fractures resulting from OP become increasingly common in women after age 55 years and men after age 65 years, resulting in the increase in bone-associated morbidity, mortality and health-care costs [2]. With the aging of the population structure, the incidence of senile osteoporosis (SOP) is on the rise, which has become an increasingly serious health problem in the world [3]. The inhibition of both osteoblastic bone formation and osteoclastic bone resorption with age, and the prevalent bone resorption that overpasses bone formation is the fundamental pathogenesis in SOP [4, 5]. At present, the treatment of SOP, as same as OP, mainly relies on calcium supplementation, inhibition of bone resorption and promotion of bone formation [6]. However, several issues can interfere with the effectiveness of anti-SOP drugs in clinical practice, such as poor adherence to therapy and safety in long-term treatment, especially for elderly patients with liver and kidney dysfunction [7]. More importantly, these drugs target osteoblastic bone formation or osteoclastic bone resorption only and nearly no drugs targeting the both [8], which will effectively reduce the bone loss with fewer side effects. Therefore, it is necessary to find new effective drugs to overcome these problems in pursuit of better effect.

Herbal medicines have been prescribed in treating SOP for a long time, and numerous investigations confirmed some herbal medicines with bone protective effects (including natural compounds and marketed prescriptions). These findings indicate that herbal medicines can affect both bone formation and bone resorption [4]. Traditional Chinese medicine (TCM) believes that SOP is mainly caused by *Shen* (Kidney) deficiency. With the increasing of age, weak body, *Shen* deficiency, bone marrow loss and bone dystrophy are the main pathogenesis of SOP. Thus, the treatment of SOP accordingly follows the rule of tonifying *Shen*. Many herbs have been proved the function of *Shen*-tonify activities in TCM formulas for preventing and treating OP with lower toxicity compared with chemical synthetic drugs [9]. Among them, *Epimedii Folium* (EF) with the function of replenishing *Shen-yang* and *Ligustri Lucidi Fructus* (LLF) of replenishing *Shen-yin* are commonly used in the clinical treatment of SOP [10]. The combined formula of EF and LLF (EF&LLF) has been applied to treat OP for almost 50 years by Professor *Shizeng Li* and authorized by the State Patent Office of China (Patent No. Zl201410037992.5). Our previous researches demonstrated that EF&LLF had an anti-osteoporosis actions on ovariectomized rats [11], glucocorticoid-induced osteoporosis (GIOP) rats [12] and retinoic acid-induced osteoporosis rats [13]. Moreover, following investigations with effective components of EF&LLF, icariin and oleanolic acid have shown the effects on bone formation and bone resorption [14, 15]. However, both the direct and indirect effects of EF&LLF on SOP as well as its underlying mechanism are rarely investigated.

The balance of bone remodeling is important for maintaining bone structure and its functional activities [16], similar to the balance of *yin* and *yang* in TCM. Thus, the treatment principle of EF&LLF for SOP, restoring the balance between *yin* and *yang* to invigorate *Shen* and strengthen bone, is similar to rebalancing the bone remodeling. Under normal physiological conditions, bone tissue is always in the dynamic balance of osteoclasts absorbing old bone and osteoblasts forming new bone [17, 18]. With the increase of age, the activity and quantity of osteoblasts and osteoclasts decrease gradually, and the function of osteoclasts is relatively enhanced [19]. Thus an ideal drug for the treatment of SOP could stimulate both bone formation and bone resorption; more importantly, its stimulation for bone formation should be superior to that of bone resorption. The purpose of this study was to investigate whether EF&LLF could restore the bone remodeling through stimulating osteoblastogenesis and osteoclastogenesis in SOP and explore the mechanism of rebalancing bone remolding.

SOP is a systemic metabolic bone disease, and its aging process is accompanied by a variety of factors related to the homeostasis of bone remodeling. A single indicator cannot comprehensively and accurately assess the progression of SOP. Therefore, it is necessary to establish an appropriate SOP prediction model for assessing the efficacy of anti-SOP drugs. Artificial neural networks (ANNs) are complex mathematical models inspired from biological neural networks, which are capable to learn and adapt their predictive algorithms after the introduction of new information [20]. Multi-layer perception (MLP) is an important nonlinear multi-factor analysis method and one of the most widely used ANN models. In our previous study [21], a credible MLP-ANN model has been designed according to the dynamic changes of osteoporosis parameters in SOP rats aged from 6 to 17 months. In the present study, we focused on exploring the changes of the parameters related to bone remodeling in SOP rats, used the constructed MLP-ANN model to evaluate and compare the therapeutic effect of EF and LLF in single use or in combination, and provide experimental basis for the treatment of clinical SOP.

## Methods

### Preparation of active ingredients

*Epimedii Folium* (EF, dried leaf of *Epimediium brevicornu Maxin*) and *Ligustri Lucidi Fructus* (LLF, dried mature seed of *Ligustrum lucidum Ait*) were purchased from Beijing *Tongrentang* pharmaceutical Co. Ltd., China. They were authenticated by an expert herbalist, *Shiyuan Jin*, Honorary Professor, School of TCM, Capital Medical University. Voucher specimens were deposited at the TCM Endocrine and Metabolic Disease Laboratory of TCM School of Capital Medical University, China. Preparation of aqueous extract of EF, LLF or EF&LLF was performed according to the methods described before [13]. And the procession of extracting combined active ingredients has been protected by the Chinese patent (20140037992.5). The extract of EF&LLF was mainly total flavonoid and total iridoid, including icariin, epimedin A, epimedin B, epimedin C, salidroside, tyrosol, specnuezhenide and ligustrosidic acid. The combination of EF and LLF were mixed at a ratio of 4 to 3 of the raw herbal according to clinical practice. Before application, the combined active fractions were dissolved with distilled water at 10 mg/mL.

### Animals

Forty male SD rats were purchased from Beijing Vital River Laboratory Animal Technology Co. Ltd. (Beijing, China), including 8 6 month old rats and 32 8 month old rats. Experiments were approved by the Ethics Committee of Capital Medical University (No. AEEI-2016-178). All the rats were housed in clear plastic cages in temperature- and light-controlled room (23 ± 2 °C and light cycle of 12 h). Food and water were available ad libitum.

### Experimental design

**Fig.1:**
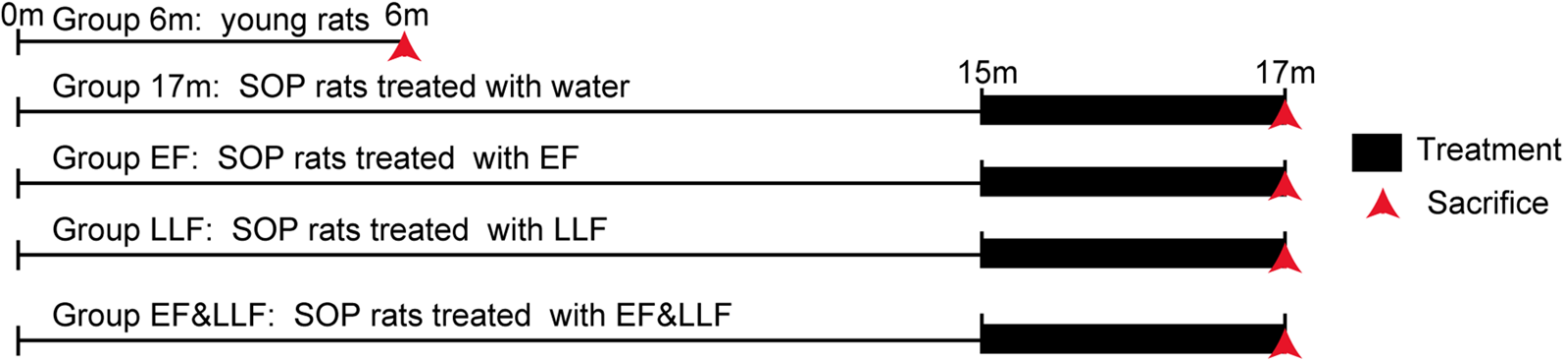
Schematic representation of the experimental design

After grew up to 15 months old, rats were randomly allocated into 4 groups, 8 rats in each group: 17 m group, EF group (2 g/kg), LLF group (1.5 g/kg) and EL&LLF group (3.5 g/kg). All of the animals were treated once daily for 8 weeks. The dose was adjusted to the body weight recorded twice a week. 6 month-old rats were sacrificed after 1 week of adaptation to the environment. When the other rats grew to 17 months old, we anesthetized them with 25% ethyl carbamate (4 mL/kg body weight) by intraperitoneal injection. After righting reaction disappeared, we nipped rat’s claws with tweezers to further determine if the anesthesia succeeded. We fixed the rat with medical proof fabric, and put its tongue on side to keep it breathing smoothly. Blood samples were collected and separated using a centrifuge (Biofuge 15 R, Heraeus Sepatech, Baxter International, Deerfield, IL, USA). Liver tissues were immediately dissected out, washed with ice-cold saline and weighed. Parts of the livers from each group were taken to be preserved in 10% formalin saline for histopathological examinations. The right femur was removed, wrapped with 0.9% saline-saturated gauze and stored at − 20 °C for the testing of mCT, BMD, bone biomechanics and bone mass. The right tibia was dissected and fixed in 4% paraformaldehyde for paraffin section. The left femur was sectioned and frozen at − 80 °C for quantitative real-time polymerase chain reaction (qPCR) and western blot analyses. There is a scheme explaining the experimental protocol in Fig. 1.

### Bone mineral density (BMD)

BMD of each right femur was measured with a dual-energy X-ray absorptiometry system (DEXA, Lexxos-2000, Medlink, France) equipped with appropriate software for bone assessment in small animals according to our previous report [21].

### Bone biomechanical testing

After BMD measurement, the biomechanical properties of the right femur were assessed using a diaphysis bending test on a WD-1 Universal Testing Apparatus (KeXin Testing Machine Co., Ltd., Changchun, China) that was equipped with calculation and analysis software according to our previous report [21]. Loading-displacement curves were recorded on-line and analyzed to determine the ultimate load and displacement at ultimate.

### Bone mass testing

When the rats were sacrificed, we weighed the right femur for wet weight, then put it in distilled water to measure its total volume. After bone biomechanical testing, we dried the right femur in an oven at 110 °C for 24 h to measure their dry weight, then burned them into ashes in a muffle at 600 °C for 3 h, and cooled it in a desiccator (SFG-02B, Hengfeng, China) for 30 min.

### Micro-computerized tomography (mCT)

The entire scan length was set for 5 mm from top of the right femur to proximal direction of the femur in a spatial resolution of 17 μm per voxel with a 3072 × 2048 image matrix by mCT system (Inveon, Siemens, Germany). 3D structures of trabecular bone were reconstructed. Bone tissue volume fraction (bone volume/total volume, BV/TV), bone surface fraction (bone surface/bone volume, BS/BV), trabecular number (Tb.N), trabecular thickness (Tb.Th), trabecular separation (Tb.Sp) and cortical wall thickness (CWT) were measured using built-in software.

### Hematoxylin–eosin (H&E) staining

Rat liver and decalcified tibias were embedded in paraffin by standard histological procedures. The stained tissues were observed and imaged with a light microscope (Nikon Eclipse 80i, Tokyo, Japan). Histomorphometry variables were analyzed using an image analyzing computer system (NIS-Elements BR 3.2, Nikon, Japan) linked to the microscope.

### Hepatic function and blood lipids

Biochemical test kits of triglyceride (TG), total cholesterol (TC), low-density lipoprotein cholesterol (LDL-C), high-density lipoprotein cholesterol (HDL-C), aspartate aminotransferase (AST), alanine aminotransferase (ALT) and γ-glutamyl transferase (GGT) were provided by InTec Products Inc. (Xiamen, China). AST, ALT, GGT, TC, TG, LDL-C and HDL-C were determined in serum samples according to the manufacturers’ instructions.

### Enzyme-linked immunosorbent assay (ELISA)

ELISA kits of rat bone-specific alkaline phosphatase (bALP, cat: E02B0138), tartrate-resistant acid phosphatase (TRACP, cat: E02T0537), osteoprotegerin (OPG, cat: E02O0020) and receptor activator of nuclear factor-κ B ligand (RANKL, cat: E02R0005) were provided by Blue Gene Biotech Co., Ltd. (Shanghai, China). Rat transforming growth factor-β1 ELISA kit (TGF-β1; cat:EK3812/2) was provided by LIANKE Biotech Co., Ltd. (Hangzhou, China). Serum bALP, TRACP, OPG, RANKL and TGF-β1 were measured by ELISA kits according to the protocols provided by manufacturers.

### Bone ALP and TRACP staining

Sections of decalcifed tibias were stained with ALP and TRACP stain kits (Nanjing Jiancheng Bioengineering Institute, Nanjing, China) for osteoblast activity and osteoclast activity. Three visual fields were measured by an image analyzing computer system (NIS-Elements BR 3.2, Nikon, Japan) linked to a microscope (Nikon Eclipse 80i, Tokyo, Japan). Integral optical density (IOD) of ALP or osteoclast number was determined from ALP-stained or TRACP-stained sections [11].

### Immunofluorescence (IF) staining

The tibia was fixed in a 10% formaldehyde solution for 24 h and then was used to make a 5 μm thick cross-section. Slices were dewaxed with xylene and dehydrated with 100 to 70% alcohol gradient. The primary antibody, insulin-like growth factor (IGF-1), was diluted with PBS at a ratio of 1:50. The FITC-fluorescent secondary antibody was diluted with PBS at a ratio of 1:100. The results of green fluorescent protein (GFP) and nuclear blue fluorescence (DAPI) were performed with NIS-Elements BR3.2 software.

### Quantitative real-time polymerase chain reaction (qPCR)

Total RNA (2ug) was extracted by Trizol reagent after pulverizing in liquid nitrogen following the manufacturer’s instructions. The target gene was reversed transcription on Bio-Rad iQ5 PCR cycler using a real-time quantitative PCR kit. The Real-time PCR reaction was then carried out according to the following reaction conditions: 95 °C, 15 min (pre-denaturation); 95 °C, 12 s (denaturation); 60 °C, 1 min (annealing); 10 s, 60 °C (extension) for a total of 40 cycles. β-actin served as an internal reference gene, and the negative control sample was a calibration sample. The primers were synthesized by Tiangen Biotech Co., Ltd. (Beijing, China), and the primer sequences were listed in Table 1.

**Table 1:** Primer used for qPCR

Primer	Upstream	Downstream	Product size
RANKL	GCAGCATCGCTCTGTTCCTGTA	GCATGAGTCAGGTAGTGCTTCTGTG	164 bp
M-CSF	GAATGACTGAACCTGCCTGCTGAA	AGGCCAGCTCAGTGCAAGAA	117 bp
Wnt5a	ACAGGCAGTGGCATGCAGA	CAGGCAGCTGTTGACCTAGGAA	134 bp
Atp6v0d2	CGAGGCATTCTACAAATTCTGCAA	TTCAGTGCCAAATGAGTTCAGAGTG	124 bp
OPG	CTCATCAGTTGGTGGGAATGAAGA	ACCTGGCAGCTTTGCACAATTA	107 bp
IGF-1	GCACTCTGCTTGCTCACCTTTA	TCCGAATGCTGGAGCCATA	148 bp
TGF-β1	CATTGCTGTCCCGTGCAGA	AGGTAACGCCAGGAATTGTTGCTA	103 bp
BMP2	ACCGTGCTCAGCTTCCATCAC	CTATTTCCCAAAGCTTCCTGCATTT	170 bp
β-actin	CACTTTCTACAATGAGCTGCG	CTGGATGGCTACGTACATGG	129 bp

*RANKL* nuclear factor-kappa B ligand, *M-CSF* macrophage colony-stimulating factor, *Atp6v0d2* d2 isoform of vacuolar (H +) ATPase, *OPG* osteoprotegerin, *IGF-1* insulin-like growth factor-1, *TGF-β1* transforming growth factor-β1, *BMP2* bone morphogenetic protein-2

### Western blot (WB)

The left femur were lysed on ice in the RIPA buffer with protease inhibitor cocktail for 40 min to extract total proteins. The proteins were analyzed with a bicinchoninic acid (BCA) protein assay kit and 30 μg/lane were used for WB analysis as previously described [21], using antibodies, including TGF-β1 (1:4000), RANKL (1:500), Wnt5a (1:4000), Atp6v0d2 (1:500), M-CSF (1:500), OPG (1:5000) and BMP-2 (1:2000), and internal reference protein of β-Tubulin (1:1000).

### Evaluatin based on MLP-ANN

In our previous study, a MLP-ANN model had been established according to the dynamic changes of relevant parameters in male rats aged 3, 6, 9, 12, 15 and 17 months using SPSS 21.0 software (SPSS Inc., Chicago, USA) [21]. The agreement between the experimental data and the predicted value of MLP is very high, and the model is credible, so we used the constructed MLP model to evaluate the comprehensive effect of EF and LLF in single use or combined use on SOP rats.

### Statistical analysis

Data were presented as mean ± standard error of the mean (*SEM*). We use the single sample K-S test in nonparametric test, and find that the sample population obeys normal distribution. Multiple groups of independent samples were compared by ANOVA analysis of SPSS 21.0. The variance homogeneity test shows that the variance of the sample population is equal and the variance is homogeneous. The least significant difference (LSD) test when the variances were equal was used for comparisons between individual groups and to determine which means differed statistically significantly (*P* < 0.05).

## Results

### EF&LLF improved bone mass and quality

Bone mass and bone quality were evaluated by bone weigh, BMD, bone biomechanics and mCT. A study has found that decreased fracture rate is associated with increased bone weight [22]. BMD is an important choice for osteoporosis assessment; the changes in BMD affect bone strength [23]. Bone biomechanics is a direct and reliable method for evaluating bone quality by studying the mechanical properties of bone under external force and the biological effects after stress [24]. mCT, a non-destructive technology that provides three-dimensional (3D) images of bones in vitro, is often used as an aid to assess bone quality from both macroscopic and microscopic perspectives [25].

**Fig. 2:**
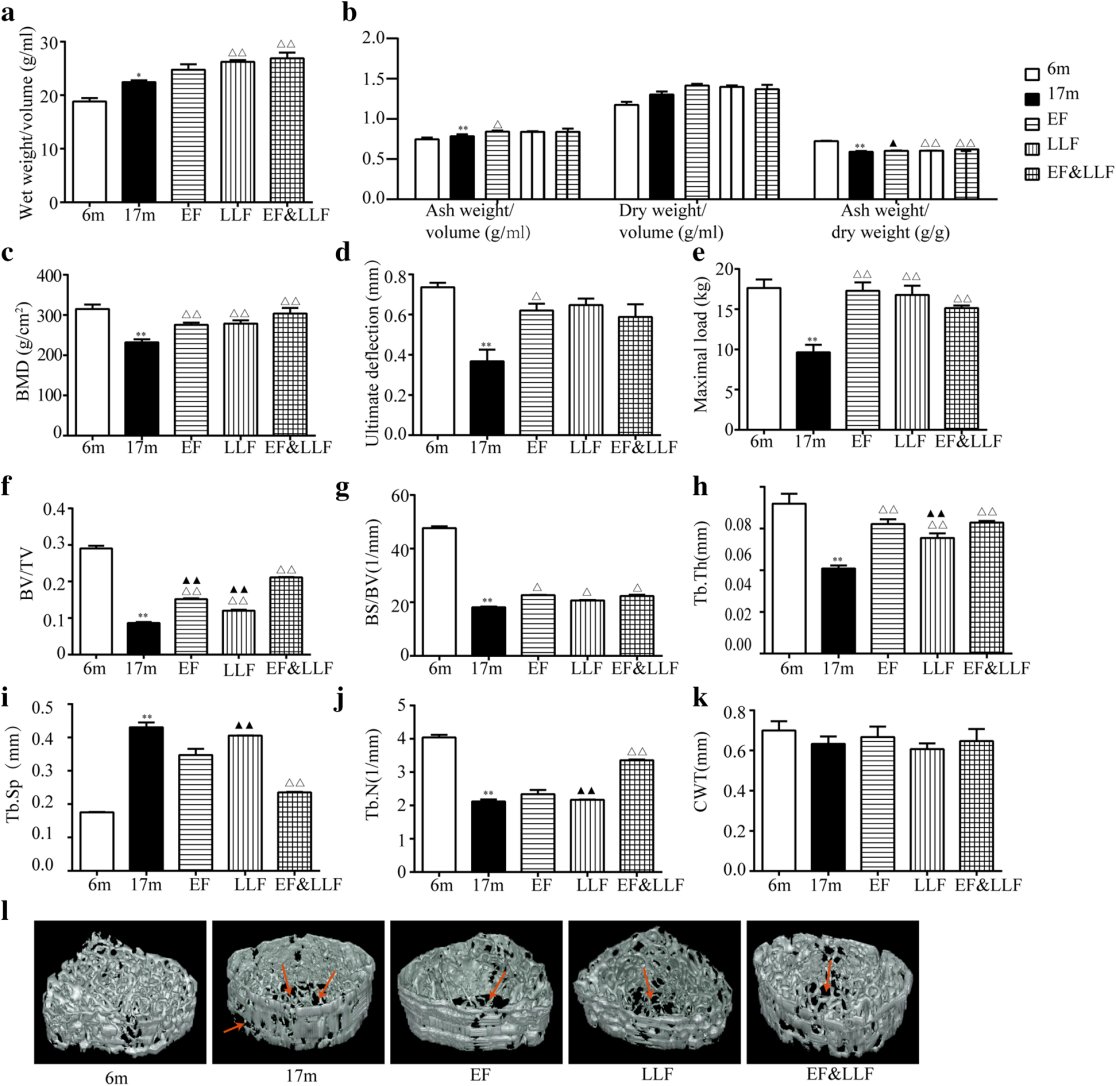
Effects of EF&LLF on bone mass and bone quality. **a**, **b** Bone weights were calculated, including the ratio of dry weight, wet weight and ash weight to bone volume and the ratio of ash weight to dry weight. **c** BMD was measured by dual-energy X-ray absorptiometry. **d**, **e** Bone biomechanical properties, including ultimate deflection and maximal load, were measured by three-point bending test. **f**–**k** Parameters of trabecular bone micro-architecture by mCT, including BV/TV (Bone Volume/Total Volume), BS/BV (Bone Surface/Bone Volume), Tb.Th (trabecular thickness), Tb.Sp (trabecular separation), Tb.N (trabecular number) and CWT (cortical wall thickness). **l** 3D images of trabecular bone micro-architecture by mCT. Mean ± SEM, *n* = 6. *P < 0.05 and **P < 0.01 vs. 6 m; ^△^*P* < 0.05 and ^△△^*P* < 0.01 vs. 17 m; ^▲^*P* < 0.05 and ^▲▲^*P* < 0.01 vs. EF&LLF

To our expectation, 17 m aged rats showed significant a low bone mass and poor bone quality demonstrated by the decreased ash weight/dry weight, BMD, ultimate deflection and maximal load (Fig. 2b-e), while the wet weight/volume and ash weight/volume were increased compared with the 6 m young rats (Fig. 2a, b); EF&LLF intervention significantly improved bone mass and quality in aged rats, manifested by increased weight/volume, ash weight/dry weight, BMD, maximal load. The mCT images of the trabecular bone micro-architecture were presented in Fig. 2l. We found that the microstructure of bone was degenerative and the trabecular structure was destroyed in 17 m aged rats compared with the 6 m young rats; EF&LLF significantly improved tightness of bone trabecular and the degree of bone connection by increasing BV/TV (Fig. 2f), BS/BV (Fig. 2g), Tb.Th (Fig. 2h) and Tb.N (Fig. 2i) as well as decreasing Tb.Sp (Fig. 2g).

Furthermore, we observed some significant difference (BV/TV, Tb.Th, Tb.Sp and Tb.N) between the EF&LLF group and LLF group (all *P* < 0.01). In addition, EF&LLF significantly increased ash weight/dry weight and BV/TV compared with the EF group (*P* < 0.05 or *P* < 0.01).

### EF&LLF ameliorated bone histopathology

**Fig. 3:**
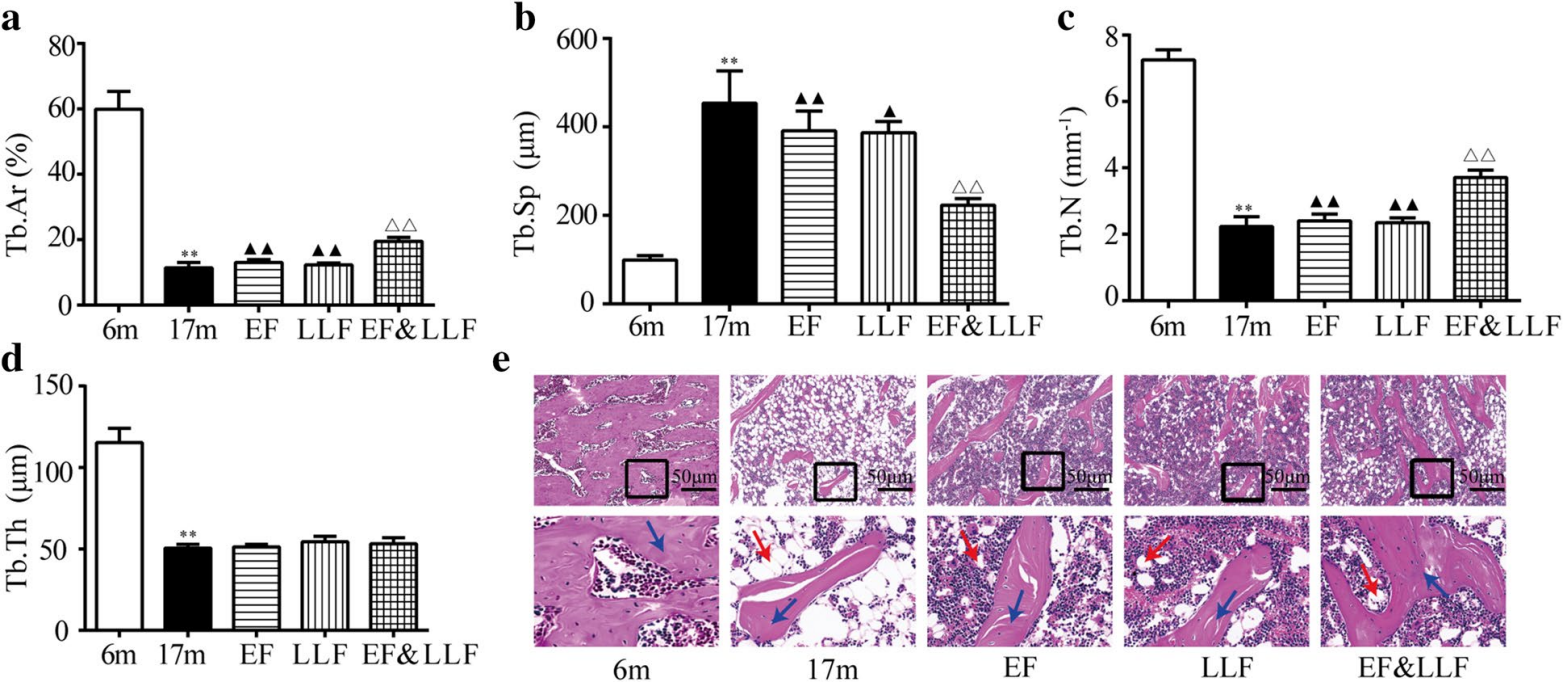
Effects of EF&LLF on bone histopathology. **a**–**d** Parameters of bone histomorphometry of the trabecular bone included Tb.Ar, Tb.Sp, Tb.N and Tb.Th. (F) Representative H&E staining images of trabecular bone from each group (× 100). Arrow (bule): trabecular bone; Arrow (red): adipocyte. Mean ± SEM, *n* = 6. **P < 0.01 vs. 6 m; ^△△^*P* < 0.01 vs. 17 m; ^▲^*P* < 0.05 and ^▲▲^*P* < 0.01 vs. EF&LLF

The alterations in the structure of trabecular bone observed by H&E staining tibia were almost in accordance with the micro-structural changes revealed by mCT. The area, number and thickness of trabecular bone were decreased while trabecular separation was increased in 17 m aged rats (Fig. 3). EF&LLF significantly increased Tb.Ar and Tb.N, and decreased Tb.Sp compared with the 17 m group (all *P* < 0.01). The effects of EF&LLF on Tb.Ar, Tb.N and Tb.Sp were significantly different from that of EF or LLF (*P* < 0.05 or *P* < 0.01). Interesting, we found that there were more fat cells in aged bone tissue, inferring that more adipocytes cause the decreased differentiation of bone cells because bone, cartilage and adipocytes were all come from mesenchymal stem cells [26]. After treatment with EF and LLF in single use or combined use, adipocytes were obviously decreased in bone pathological section compared with 17 m group.

### Effects of EF&LLF on body weight, blood lipids and liver function

**Fig. 4:**
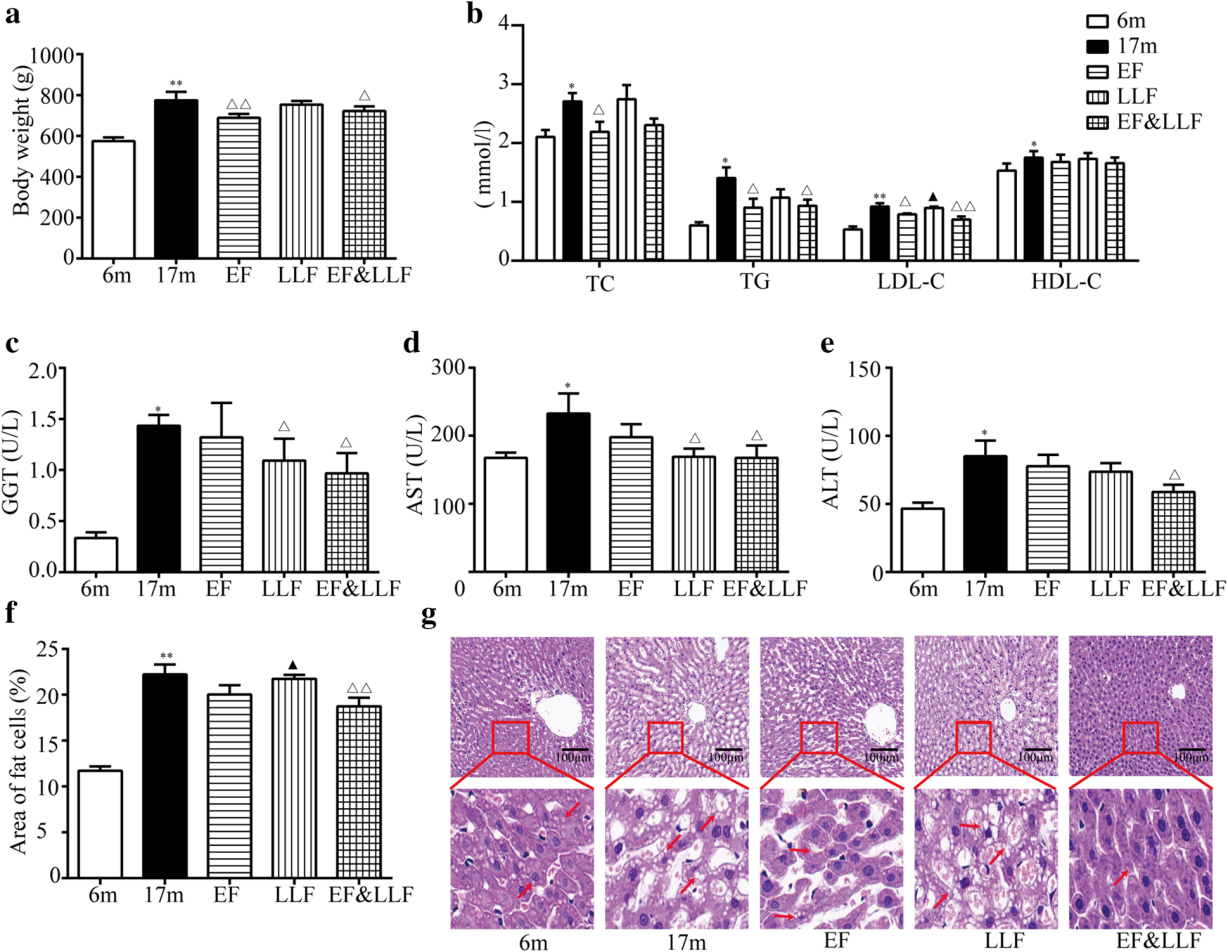
Effects of EF&LLF on body weight, blood lipids and liver function. **a** Body weight. **b** Levels of total cholesterol (TC), triglyceride (TG), low density lipoprotein cholesterol (LDL-C) and high density lipoprotein cholesterol (HDL-C) in serum were measured by colorimetric kits. **c**–**e** Serum γ-glutamyl transferase (GGT), aspartate-aminotransferase (AST) and alanine-aminotransferase (ALT) were measured by colorimetric kits. **f** Area of adipocytes (%) in liver was calculated. **g** Representative photographs of H&E-stained liver sections from each group (× 200). Arrow (red): adipocytes. Mean ± SEM, *n* = 6 or 8. *P < 0.05 and **P < 0.01 vs. 6 m; ^△^*P* < 0.05 and ^△△^*P* < 0.01 vs. 17 m; ^**▲**^*P* < 0.05 vs. EF&LLF

Gain of fat mass is significantly and independently associated with declining physical performance as well as an increased risk for disability, hospitalizations, and mortality in older individuals. This increased fat mass is not exclusively stored in adipose depots but may become deposited in non-adipose tissues [27]. Our study found that a significant weight gain (Fig. 4a) and the significant elevation of TG, TC, LDL-C and HDL-C levels in 17 m aged rats compared with 6 m rats (Fig. 4b). After administration of EF, LLF or EF&LLF, EF and EF&LLF highly decreased body weight and the elevated levels of TG and LDL-C in comparison with the 17 m group; EF reduced TC level. The effect of EF&LLF on LDL-C was significantly different from that of LLF (*P* < 0.05).

The liver plays essential roles in metabolism, toxicants clearance, regulation of inflammation, and molecule biosynthesis. A study demonstrates the aged rats have higher hepatic lipid accumulation and hepatic cell death as compared with young rats [28]. Figure 4c-e showed the effects of EF&LLF on hepatic function. ALT, AST and GGT levels were significantly elevated in 17 m aged rats in comparison to 6 m rats. In contrast, treatment with EF&LLF normalized all the elevated liver function parameters; treatment with LLF decreased GGT and ALT levels. Liver histopathological examination of 17 m group revealed the increased area of adipocyte compared with the 6 m group. EF&LLF ameliorated hepatic lipid accumulation in 17 aged rats (Fig. 2f, g). The effect of EF&LLF on the area of adipocytes in liver was significantly different from that of LLF (*P* < 0.05).

### EF&LLF regulated bone turnover rate

**Fig. 5:**
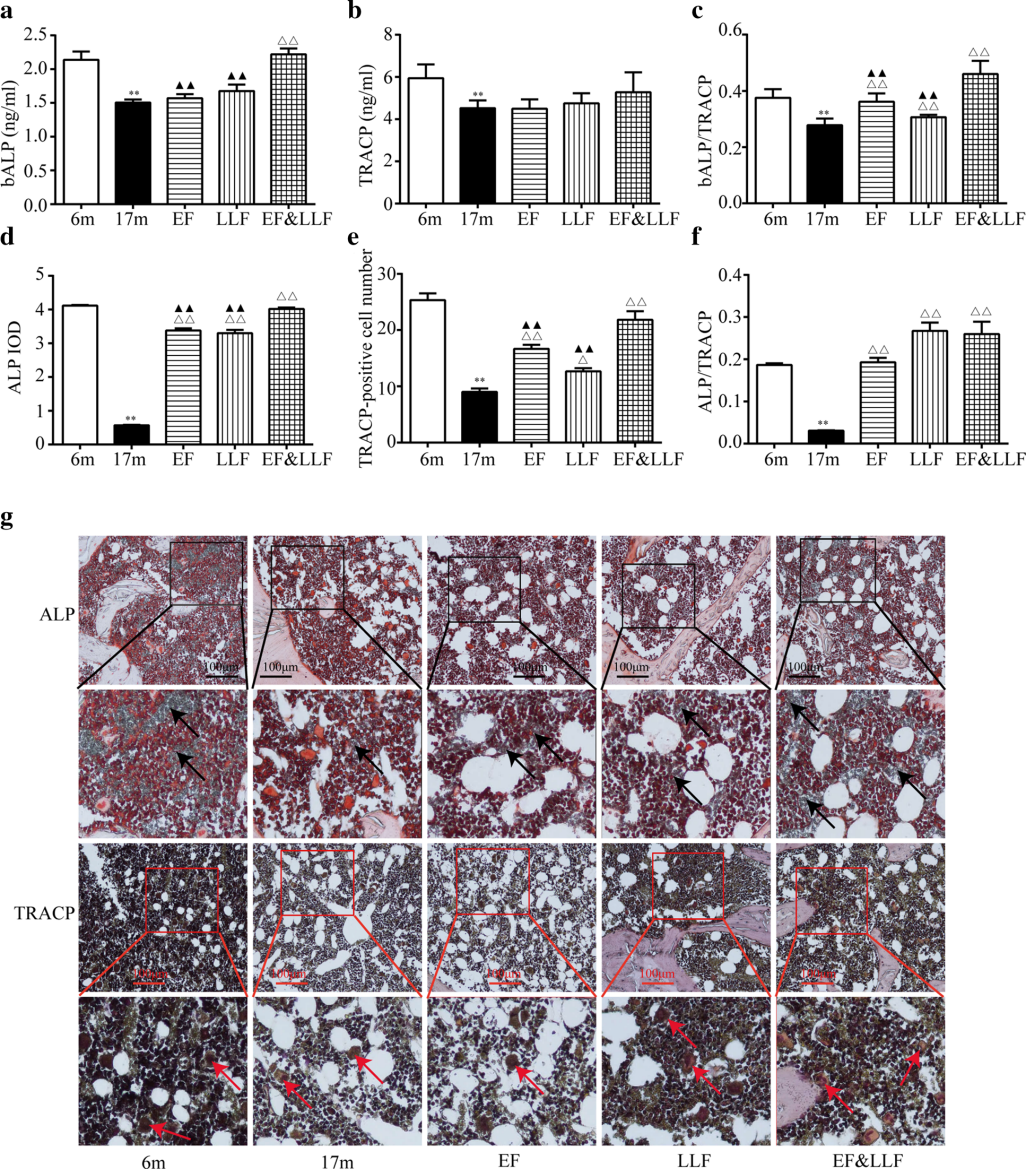
EF&LLF regulated bone turnover rate. **a**, **b** Serum bALP and TRACP were measured by ELISA. **c** bALP/TRACP in serum was calculated. **d**, **e** Activity of ALP or TRACP in tibia was measured using ALP or TRACP stain kit. **f** ALP/TRACP in tibia was calculated. **g** Representative photographs of ALP and TRACP staining were showed (× 200). Arrow (black): ALP-stained positive cell (osteoblast). Arrow (red): TRACP-stained positive cell (osteoclast). Mean ± SEM, *n* = 6 or 8. **P < 0.01 vs. 6 m; ^△^*P* < 0.05 and ^△△^*P* < 0.01 *vs.* 17 m; ^▲▲^*P* < 0.01 vs. EF&LLF

ALP is synthesized and stored in the cytoplasm of osteoblasts, which is a specific index reflecting bone formation. TRACP is derived from osteoclasts and is a specific index reflecting bone resorption. ALP/TRACP can be used in evaluating bone turnover rate or bone remodeling [29]. We observed that bALP, TRACP and bALP/TRACP in serum and ALP, TRACP and ALP/TRACP in bone were significantly decreased in 17 m aged rats compared with 6 m rats (Fig. 5 or *P* < 0.01), suggesting the changes in bone remodeling resulting in a net bone loss. Compared with the 17 m group, only EF&LLF significantly increased serum bALP and bone TRACP; EF, LLF and EF&LLF raised bone ALP and ALP/TRACP in serum and bone (*P* < 0.05 or *P* < 0.01). Furthermore, we observed some significant difference (serum bALP, serum bALP/TRACP, bone ALP and bone TRACP) between EF&LLF group and EF or LLF group (all *P* < 0.01). Taken together, these results indicated that EF&LLF exerted direct regulatory effects on osteoclastogenesis and osteoblastogensis and regulated bone turnover rate.

### EF&FLL upregulated the expression of TGF-β1, BMP2, Wnt5a and IGF-1

**Fig. 6:**
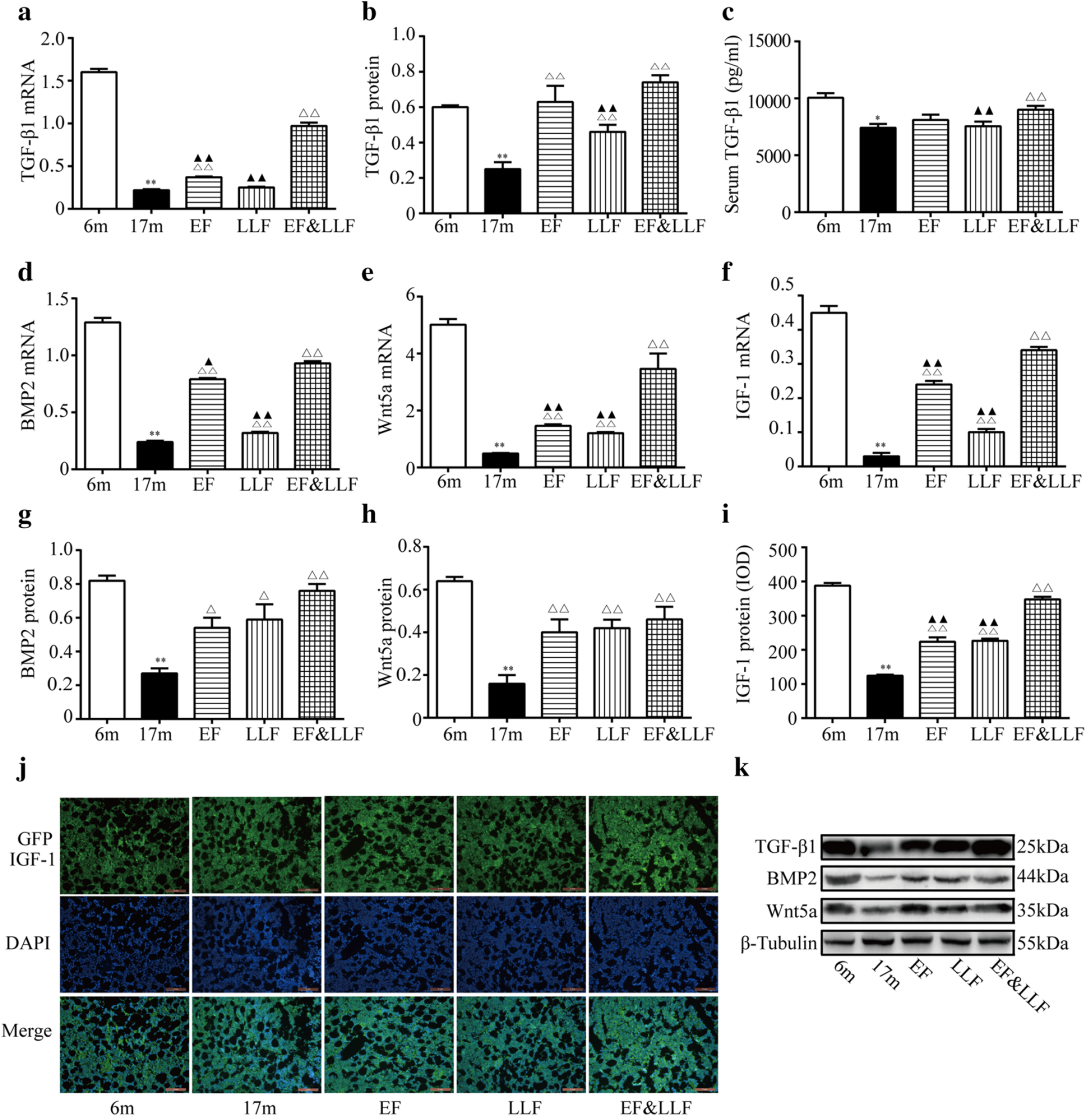
EF&FLL up-regulated the expression of TGF-β1, BMP2, Wnt5a and IGF-1. **a** TGF-β1 mRNA was measured by qPCR analysis with β-actin as an internal control. **b** TGF-β1 protein was measured by WB. **c** serum TGF-β1 was measured by ELISA. **d**–**f** mRNA of BMP2, Wnt5a and IGF-1 was measured by qPCR with β-actin as an internal control. **g**–**h** protein of BMP2 and Wnt5a was measured by WB. **i** IGF protein was measured by IF and IGF-1 IOD were calculated. **j** Representative photographs of IGF-1 from IF-stained tibia sections (× 200). **k** Representative WB photographs of TGF-β1, BMP2 and Wnt5a were viewed, and β-Tubulin was used for normalization. Mean ± SEM, *n* = 6. *P < 0.05 and **P < 0.01 vs. 6 m; ^△^*P* < 0.05 and ^△△^*P* < 0.01 vs. 17 m; ^▲^*P* < 0.05 and ^▲▲^*P* < 0.01 vs. EF&LLF

In bone remodeling cycle, reversal phase (osteoblasts are recruited to bone tissue and osteoclasts start apoptosis) and formation phase (osteoblasts produce collagen and form new bone) promote the formation of new bone [30]. TGF-β1 can promote the proliferation and differentiation of osteoblasts, induce osteoclast apoptosis and improve bone formation [31]. BMP2 is a requirement for bone formation and the product of osteoblasts synthesized and secreted into the bone matrix [32]. As one of the major growth factors involved in cartilage matrix synthesis and repair, IGF-1 promotes the synthesis of collagen type II, proteoglycans, and other matrix components [33]. Wnt5a has opposing effects on bone remodeling that are dependent on the cell of origin: osteoblast-derived Wnt5a promotes osteoclastogenesis; osteoclast-derived Wnt5a promotes bone proliferation and bone formation [34]. To investigate the mechanism of EF&LLF in affecting osteoblastic bone formation, we measured the protein and mRNA expressions of TGF-β1, BMP2, Wnt5a and IGF-1 (Fig. 6). We found that the bone mRNA and protein expressions of TGF-β1, BMP2, Wnt5a and IGF-1 in 17 m group were down-regulated compared with 6 m group (all *P* < 0.01). The alteration of serum TGF-β1 was in accordance with its changes in bone. EF&LLF promoted the formation of new bone by raising the mRNA and protein of TGF-β1, BMP2, Wnt5a and IGF-1. In addition, there are remarkable differences in IGF-1 protein and the mRNA expression of TGF-β1, BMP2, Wnt5a and IGF-1 between EF&LLF and EF or LLF (*P* < 0.05 or *P* < 0.01); EF&LLF significantly increased bone TGF-β1 protein and serum TGF-β1 level in 17 m rats, compared with that in the LLF group ( *P* < 0.01).

### EF&LLF upregulated OPG, RANKL, ATP6v0d2 and M-CSF

**Fig. 7:**
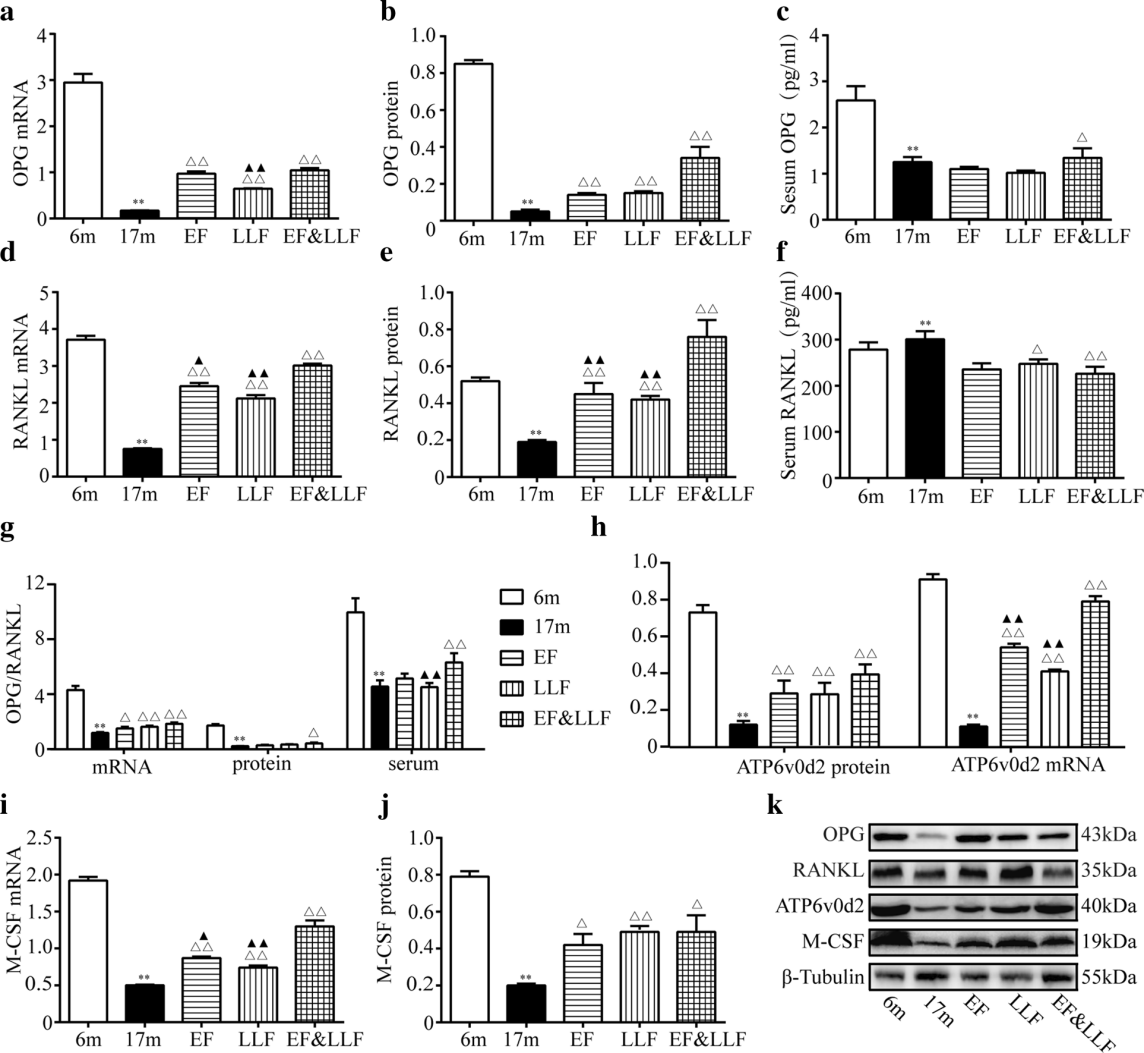
EF&FLL up-regulated the expression of OPG, RANKL, ATP6v0d2 and M-CSF. **a** Bone OPG mRNA was by qPCR analysis with β-actin as an internal control. **b** Bone OPG protein was measured by WB. **c** Serum OPG was by ELISA. **d** Bone RANKL mRNA was by qPCR. **e** Bone RANKL protein was measured by WB. **f** Serum RANKL was tested by ELISA. **g** OPG/RNAKL in bone mRNA, bone protein and serum were calculated. **h** mRNA and protein of ATP6v0d2 was measured by qPCR and WB, respectively. **i** M-CSF mRNA was tested by qPCR. **j** M-CSF protein was measured by WB. **k** Representative WB photographs of OPG, RANKL, ATP6v0d2 and M-CSF were viewed, and β-actin or β-Tubulin was used for normalization. Mean ± SEM, *n* = 6. *P < 0.05 and **P < 0.01 vs. 6 m; ^△^*P* < 0.05, ^△△^*P* < 0.01 vs. 17 m; ^▲^*P* < 0.05, ^▲▲^*P* < 0.01 vs. EF&LLF

In the bone remodeling cycle, bone resorption refers to activation phase (osteoclasts are recruited to bone tissue) and resorption phase (osteoclasts dissolute and absorb bone mineral). Differentiation of osteoclasts needs two essential conditions: M-CSF and RANKL combined with their receptor activators, respectively [35]. OPG, secreted by osteoblasts, is the endogenous decoy receptor of RANKL with the function of inhibiting osteoclast activation and promoting osteoclast apoptosis. The proportion of RANKL/OPG is a key factor in regulating bone resorption [36]. Highly expressed in osteoclasts, ATP6v0d2 may influence osteoclast maturation instead of differentiation [37]. To investigate the mechanism of EF&LLF in affecting osteoclastic bone resorption, we measured the protein and mRNA expression of OPG, RANKL, ATP6v0d2 and M-CSF (Fig. 7). Our results demonstrated that bone mRNA and protein of OPG, RANKL, ATP6v0d2 and M-CSF in the 17 m group were down-regulated compared with the 6 m group (*P* < 0.01), inferring the depressed osteoclasts function in aged rats. The alterations of serum OPG was in accordance with its changes in bone. The ratio of OPG/RANKL in bone mRNA, bone protein and serum were obviously decreased in 17 m rats compared with 6 m rats, suggesting the inhibition of osteoblasts is more serious than osteoclasts. 17 m rats treated by EF&LLF displayed higher mRNA and protein expression of OPG, RANKL, ATP6v0d2 and M-CSF. Moreover, higher OPG/RANKL in bone mRNA, bone protein and serum in EF&LLF revealed that the improvement of bone formation is better than bone resorption. In addition, EF&LLF significantly increased bone RANKL protein and the mRNA expression of RANKL, ATP6v0d2 and M-CSF, compared with EF or LLF (*P* < 0.05 or *P* < 0.01); EF&LLF significantly increased bone OPG mRNA and serum OPG/RANKL, compared with LLF (*P* < 0.01).

### Evaluation of the effects of EF&LLF based on MLP model

**Fig. 8:**
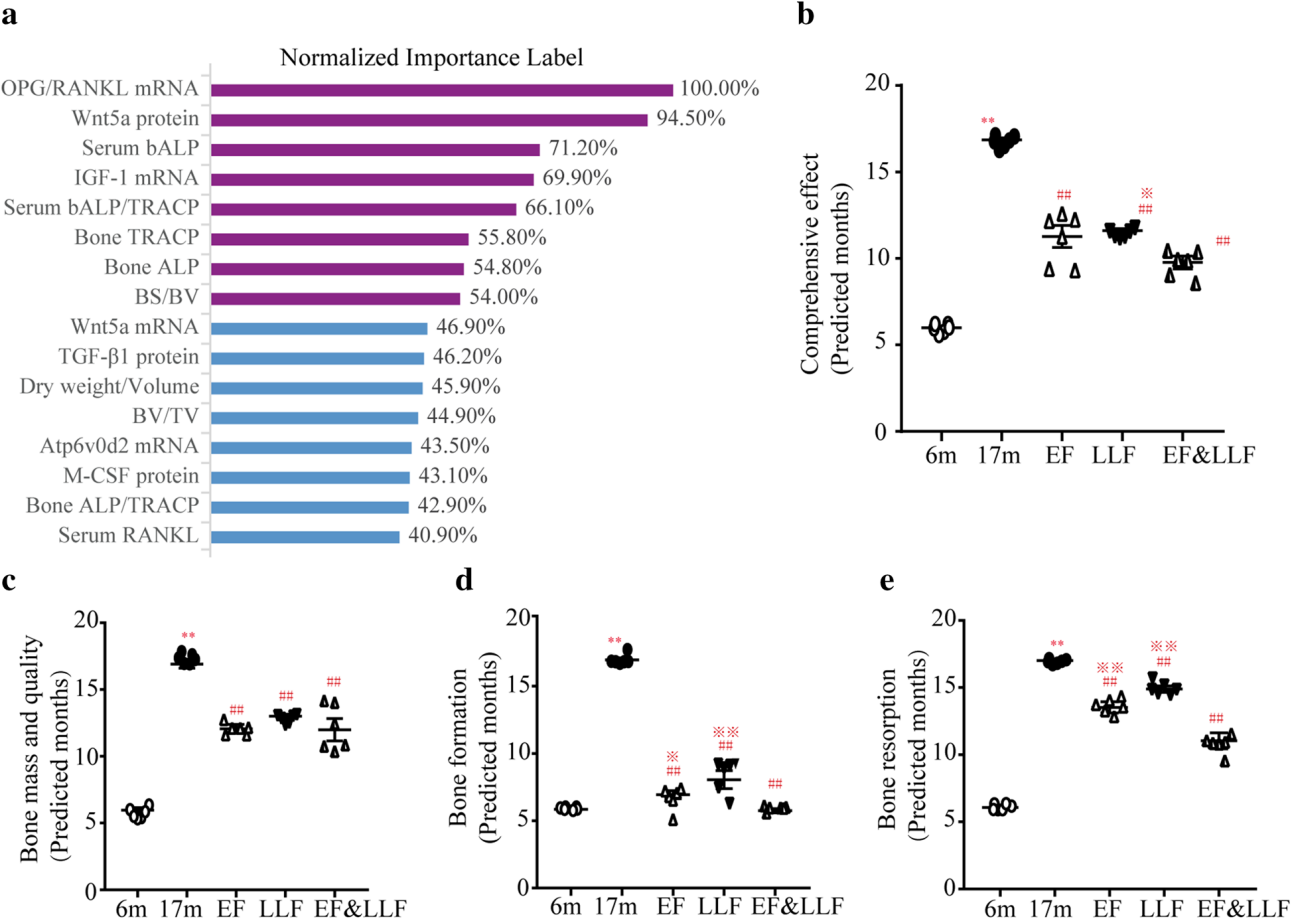
Evaluation of the effects of EF&LLF based on MLP model. The 3-layer neural network model was constructed with all the parameters in 3–17 m rats as the input layer. **a** Importance of osteoporosis parameters in aging rats. **b** Predicted months in each group based on the established MLP model according to all parameters in aging rats. **c**–**e** Predicted months in each group based on the constructed MLP model according to the parameters related to bone mass and quality, bone formation and bone resorption, respectively. Predicted values are represented as mean ± SEM. **P < 0.01 vs. 6 m; ^##^*P* < 0.01 vs. 17 m; ^※^*P* < 0.05 and ^※※^*P* < 0.01 vs. EF&LLF.  Parameters with the normalized importance of more than 50%

Using the established MLP model (Additional file 1), we assessed the importance of 41 related parameters. The top 8 (normalized importance over 50%) were OPG/RNAKL in mRNA level (100%), Wnt5a protein (94.5%), serum bALP, IGF-1 mRNA, serum bALP/TRACP, bone TRACP, bone ALP and BS/BV (Fig. 8a). Then we substituted the relevant parameters of 6, 17 m and three administration groups into the established MLP model to predict and compare the comprehensive effect of drugs. The results showed that the predicted months of the 6 m group and 17 m group were 6.00 ± 0.08 and 16.86 ± 0.12, respectively, which were close to their real situation (Fig. 8b). The aged rats treated by EF, LLF or EF&LLF recovered to a mean value of 11.27, 11.60 or 9.77 months. Compared with the 17 m group, there were significant statistical differences in the EF, LLF and EF&LLF groups. In addition, the predicted months were obviously decreased in the EF&LLF group compared with the LLF group.

The detection parameters were divided into 3 subgroups, including bone mass and quality (bone weight, BMD, bone biomechanics, mCT and bone H&E stain), bone formation (ALP, TGF-β1, BMP2, Wnt5a and IGF-1) and bone resorption (TRACP, OPG, RANKL, ATP6v0d2 and M-CSF). Then, we constructed the MLP models of 3 subgroups to evaluate the advantages of drugs. Figure 8c, e showed that EF and LLF in single use or in combined use improved bone mass and quality, bone formation and bone resorption, and what's more, EF&LLF had better effects on bone formation and bone resorption than EF or LLF.

## Discussion

EF and LLF documented in Chinese ancient medicinal literature have strong actions of replenishing Kidney-*yang* and Kidney-y*in,* respectively. They have been used to strengthen bone and treat osteoporosis for a long time in China. Osteoporosis is a chronic and complex disease that needs long-term medication intervention. However, neither *Yang*-tonifying prescription with the property of warm dryness nor *yin*-tonifying prescription with the sticky and greasy property is suitable for long-term clinical application. According to TCM theories, the combination of EF and LLF, a *Yang*-tonifying herb matched with adequate a *Yin*-tonifying herb, can make living things freely grow, flourish, and eliminate the side effects of each other [12]. EF is a famous Chinese edible herb, and the safety associated with this herb has attracted a great deal of attention due to its potential hepatotoxic effects [38]. Liver injury caused by Chinese patent medicine preparations containing EF has been frequently reported in recent years [39]. LLF is a well-known invigorator in TCM with hepatoprotective effect. The hepatoprotective effect of oleanolic acid (mainly active ingredient of LLF) was first reported in China and it has been used to treat liver disease in humans [40]. Several recent studies have supported the association between low BMD and alcoholic or nonalcoholic fatty liver disease, confirming a low negative correlation between liver fat content and lumbar BMD in middle-aged and elderly people [41, 42], and fatty liver and BMD may be linked by IGF-1, OPG, OCN, inflammatory cytokines and other factors [43]. The production of cytokines such as IL-1, IL-6, TNF-α, and RANKL during the onset of liver injury promotes osteoclast absorption, reduces BMD, and increases the risk of fracture. Alcohol has direct and indirect effects on the number and activity of osteoblasts and osteoclasts. Alcoholic fatty liver leads to imbalance of bone remodeling and reduction of bone formation. AST is normally present in tissues and cells, but low in serum. When liver cells degenerate, the permeability of cell membrane will increase, resulting in the increase of serum AST. The content of ALT is the highest in liver, which can reflect the damage of hepatocyte parenchyma. GGT is a kind of microsomal enzyme, which mainly exists in the intrahepatic bile duct epithelium and the cytoplasm of hepatocytes. High level of liver enzymes GGT, ALT and AST are important indicators to reflect liver injury [44]. In this study, an increase in serum AST, ALT and GGT, high blood lipids and hepatic lipid accumulation had been presented in 17 m rats compared with 6 m rats, suggesting that taking drugs for SOP should prevent liver injury. LLF relieved liver injury shown by the significant decrease in serum AST and GGT, while EF regulated lipid metabolism shown by the decreased TC, TG, LDL-C and area of liver adipocyte compared with 17 m rats. With the above two functions of preventing liver injury and regulating lipid metabolism, EF&LLF is more suitable for long-term use than EF and LLF.

The negative balance of bone remodeling is the basis pathogenesis of osteoporosis. Bone metabolism in the human body is a dynamic equilibrium process that is primarily regulated by bone resorption and bone formation [37]. During bone remodeling, bone resorption initiated by osteoclasts marks the beginning of the entire bone remodeling cycle, and bone formation led by osteoblast marks the end of the cycle. ALP is used commonly and clinically as a sensitive marker to reflect bone turnover rate because it can be stable and specific to represent the activity of osteoblasts [45]. As the most characteristic enzyme of osteoclasts, TRACP can reflect the activity of osteoclasts and bone resorption in vivo by detecting TRACP in serum and bone, and play a role in predicting bone quality and reducing fracture occurrence [46, 47]. ALP/TRACP can reflect the bone remolding rate. Our findings confirmed the ALP activity, ALP/TRACP and TRAP-positive osteoclasts were decreased in 17 m aged rats, which were largely increased by EF&LLF treatment. It can be inferred that both the osteoclast activity and osteoblast activity were suppressed, and the activity of osteoblasts are more weakened than that of osteoclasts in aged rats. Thus, the uncompensated bone formation to bone resorption could lead to bone loss. The protective action of EF&LLF was related to stimulating the activity of osteoblasts and osteoclasts and maintaining the balance of bone remolding in SOP rats.

**Fig.9:**
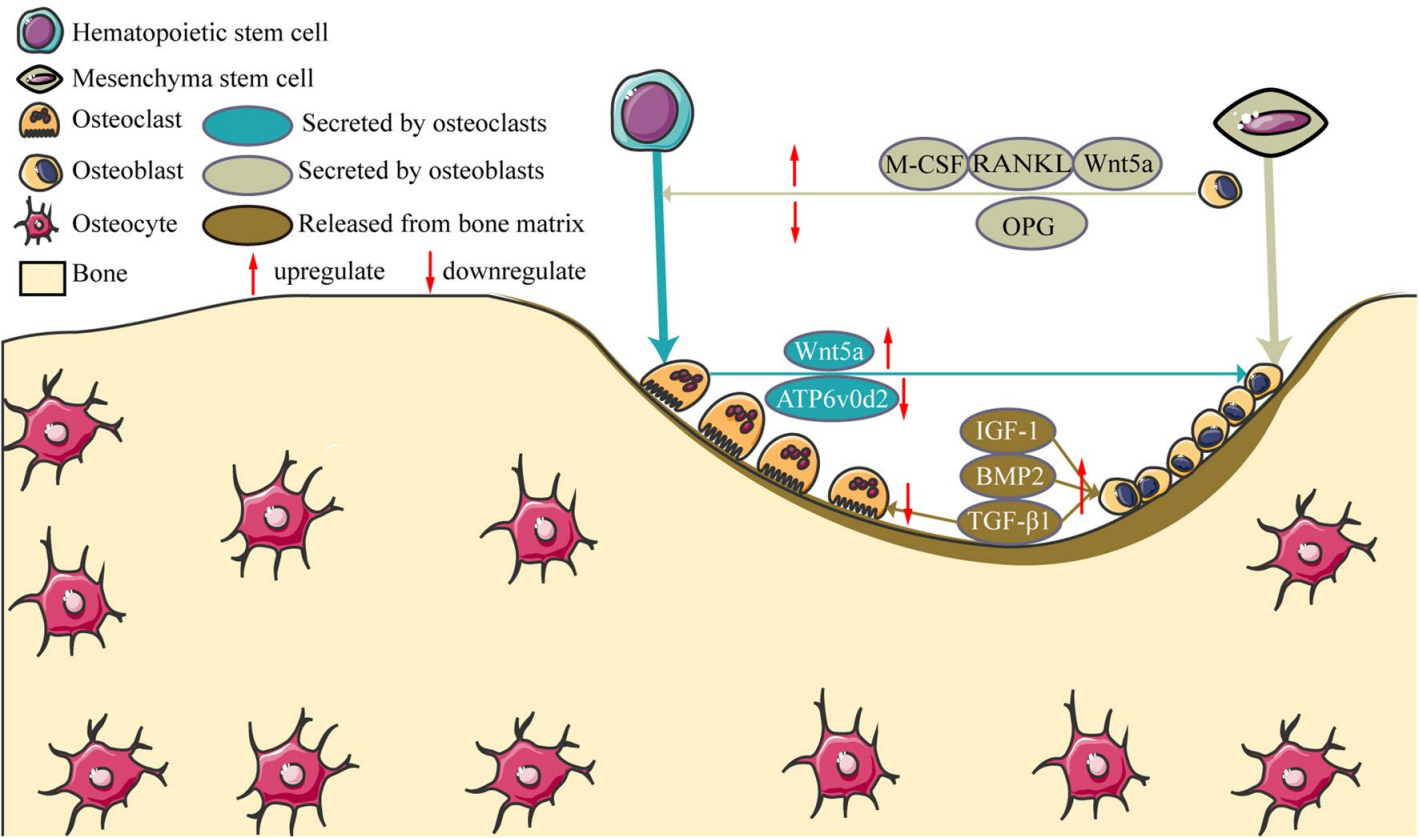
The schematic presentation of the molecular mechanisms of osteoblast-osteoclast interaction

The disordered communication between osteoclasts and osteoblasts are critical mechanisms of the imbalance of bone remodeling and bone loss. The molecular mechanisms of cellular communication between osteoblast and osteoclast are one of the central issues in bone cell biology [48]. Secreted by osteoblasts, M-CSF is an important cytokine for survival, differentiation, cell migration and activity in macrophages and osteoclasts [49], can enhanced the differentiation of bone marrow precursors to osteoclast precursors [50]. Also secreted by osteoblasts, OPG can bind with RANKL and block its bind and activation with RANK to inhibit osteoclast differentiation and activation; RANKL can bind with its receptor RANK, which is expressed on osteoclastic progenitor cells, and activates the down signaling pathways related with osteoclast growth and differentiation. The ratio of OPG to RANKL critically influences the osteoclast formation [37]. Conversely, osteoclasts also influence bone formation by osteoblasts via ATP6v0d2. Secreted by osteoclasts, ATP6v0d2, as a regulator of osteoclast fusion and bone formation, is required for efficient preosteoclast fusion and mediates extracellular acidification in bone resorption [51, 52]. Wnt5a has opposing effects on bone remodeling that are dependent on the cell of origin: osteoblast-derived Wnt5a stimulate the development of osteoclasts by up-regulating the expression of RANK [53, 54]; osteoclast-derived Wnt5a promotes bone proliferation and bone formation [34]. In addition, cytokine released from the resorbed bone matrix, such as TGF-β1, BMP2 and IGF-1, also affects the activity of osteoblasts and osteoclasts. During bone formation, BMP2 exhibits strong osteosynthetic activity in vivo and in vitro [55], and conducts information between cells and interstitial cells through autocrine and paracrine regulation, regulating the differentiation and proliferation of bone cells [56]. TGF-β1 stimulates the proliferation and differentiation of mesenchymal cells, promotes the proliferation of osteoblasts, chondrocytes and the synthesis of extracellular matrix and inhibits the production and biological activity of osteoclasts [57, 58]. IGF-1, formed by paracrine and autocrine, exists widely in bone tissue. It, playing roles in bone cell function and bone metabolism, can promote osteoblast differentiation and proliferation and inhibit withering, which is beneficial to the mineralization and synthesis of bone matrix [59]. In summary, the molecular mechanisms of cellular communication between osteoblast and osteoclast are crucial for bone remodeling. The schematic presentation of the molecular mechanisms of osteoblast-osteoclast interaction was shown in Fig. 9.

In this study, we found that the protein and mRNA expression of OPG, RANKL, Wnt5a, M-CSF, ATP6v0d2, BMP2, IGF-1 and TGF-β1, and the ratio of OPG/RANKL were significantly decreased in 17 m aged rats compared with 6 m young rats, which further demonstrated both bone formation and bone resorption were suppressed, and the uncompensated bone formation to bone resorption led to bone loss in SOP. Therefore, ideal drugs of SOP can not only stimulate osteoblastogenesis and osteoclastogenesis, but also stimulate higher osteoblastogenesis than osteoclastogenesis. And these drugs can alleviate bone loss and promote the new bone formation through taking systematic effects on both bone formation and bone resorption. EF&LLF could upregulate the protein and mRNA expression of OPG, RANKL, Wnt5a, M-CSF, ATP6v0d2, BMP2, IGF-1 and TGF-β1 and increase OPG/RANKL compared with 17 m rats. These results provided reliable evidence that EF&LLF could prevent bone loss in SOP rats through systematic regulatory effects on osteoclastogenesis and osteoblastogensis so as to stimulate higher bone turnover rate. However, further research about how these molecules affect osteoblast-osteoclast interaction is needed.

Our previous study demonstrated that the degeneration of bone structure and bone metabolism in SOP rats during the aging process of rats aged from 3 to 17 months [21]. Based on the dynamic changes of the detected indexes in male rats of different ages, such as bone mass, bone biomechanics, bone microstructure, serum bone turnover markers and bone turnover rate, we has constructed a reliable and reasonable MLP model for evaluating and comparing the comprehensive effects of drugs on treating SOP. In this study, we input the relevant parameters of 6 m and 17 m group rats into the established MLP model, and found that the agreement between the experimental data and the predicted value of MLP is very high, suggesting that the model is credible. Then, we used the MLP model to evaluate the therapeutic effect of EF and LLF on SOP rats. 17 month old rats treated with EF, LLF or EF&LLF recovered to a mean value of 11.27, 11.60 and 9.77 months, respectively, confirming that EF and LLF in single use or combined use have the osteoprotective effect. In addition, EF&LLF had better effects on bone formation and bone resorption than EF or LLF.

## Conclusion

In summary, our study provided reliable evidence that EF&LLF with fewer side effects prevented bone loss in SOP rats through taking systematic regulatory effects on osteoclastogenesis and osteoblastogensis. These findings suggested that herbal medicines might be potential resources for developing new drugs development for SOP.

## Supplementary information

**Additional file 1.** The established multilayer perception (MLP)-artificial neural network (ANN) model.

## Data Availability

The data used and/or analyzed during the current study are available from the corresponding author on reasonable request.

## Abbreviations

ALP: Alkaline phosphatase
ALT: Alanine aminotransferase
ANN: Artificial neural network
AST: Aspartate aminotransferase
ATP6v0d2: D2 isoform of vacuolar (H +) ATPase
bALP: Bone specific alkaline phosphatase
BMD: Bone mineral density
BMP2: Bone morphogenetic protein-2
BS/BV: Bone surface fraction (bone surface/bone volume)
BV/TV: Bone tissue volume fraction (bone volume/total volume)
CWT: Cortical wall thickness
EF: Epimedii Folium
EF&LL: The combined formula of EF and LLF
ELISA: Enzyme-linked immunosorbent assay
GFP: Green fluorescent protein
GGT: γ-glut amyl transferase
HDL-C: High density lipoprotein cholesterol
H&E: Hematoxylin–eosin stain
IF: Immunofluorescence
IGF-1: Insulin-like growth factor-1
IOD: Integral optical density
LDL-C: Low density lipoprotein cholesterol
LLF: Ligustri Lucidi Fructus
M-CSF: Macrophage colony-stimulating factor
mCT: Micro-Computerized Tomography
MLP: Multi-layer perception
OP: Osteoporosis
OPG: Osteoprotegerin
qPCR: Quantitative real-time polymerase chain reaction
RANKL: Receptor activator of nuclear factor-κ B ligand
SEM: Standard error of mean
SOP: Senile osteoporosis
Tb.N: Trabecular number
Tb.Sp: Trabecular separation
Tb.Th: Trabecular thickness
TC: Total cholesterol
TCM: Traditional Chinese medicine
TG: Triglyceride
TGF-β1: Transforming growth factor-β1
TRACP: Tartrate-resistant acid phosphatase
WB: Western blotting
