# Supplementary material for: Systemic osteoprotective effects of Epimedii Folium and Ligustri Lucidi Fructus in senile osteoporosis rats by promoting the osteoblastogenesis and osteoclastogenesis based on MLP-ANN model
Source: Chin Med. 2020 Aug 20;15:87. doi: 10.1186/s13020-020-00368-0 (PMC7441627; doi:10.1186/s13020-020-00368-0)
Supplement: Supplementary file 1 — Additional file 1. The established multilayer perception (MLP)-artificial neural network (ANN) model. [file 13020_2020_368_MOESM1_ESM.pdf]

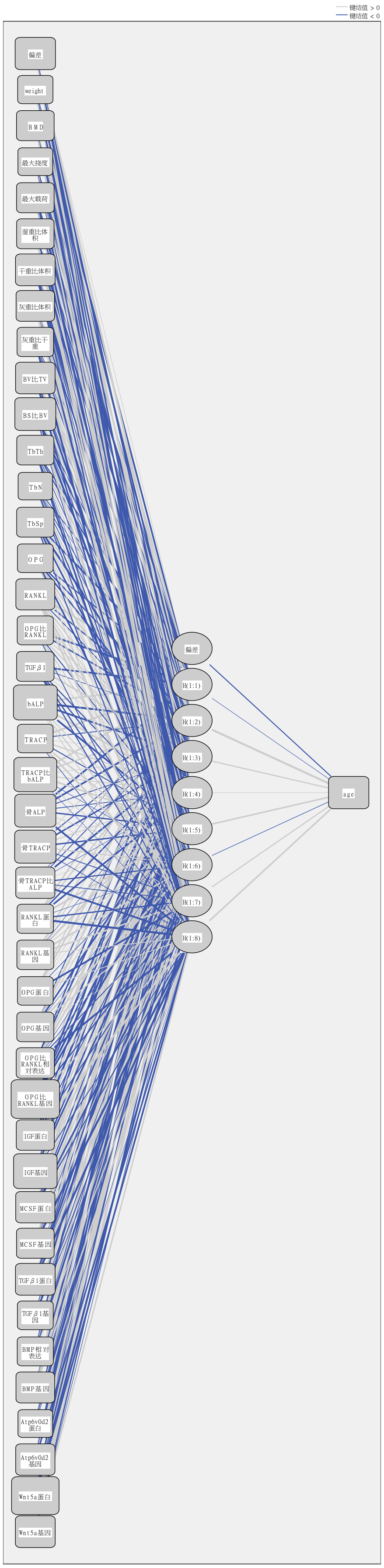

隐藏层激活函数： 双曲正切

输出层激活函数： 恒等

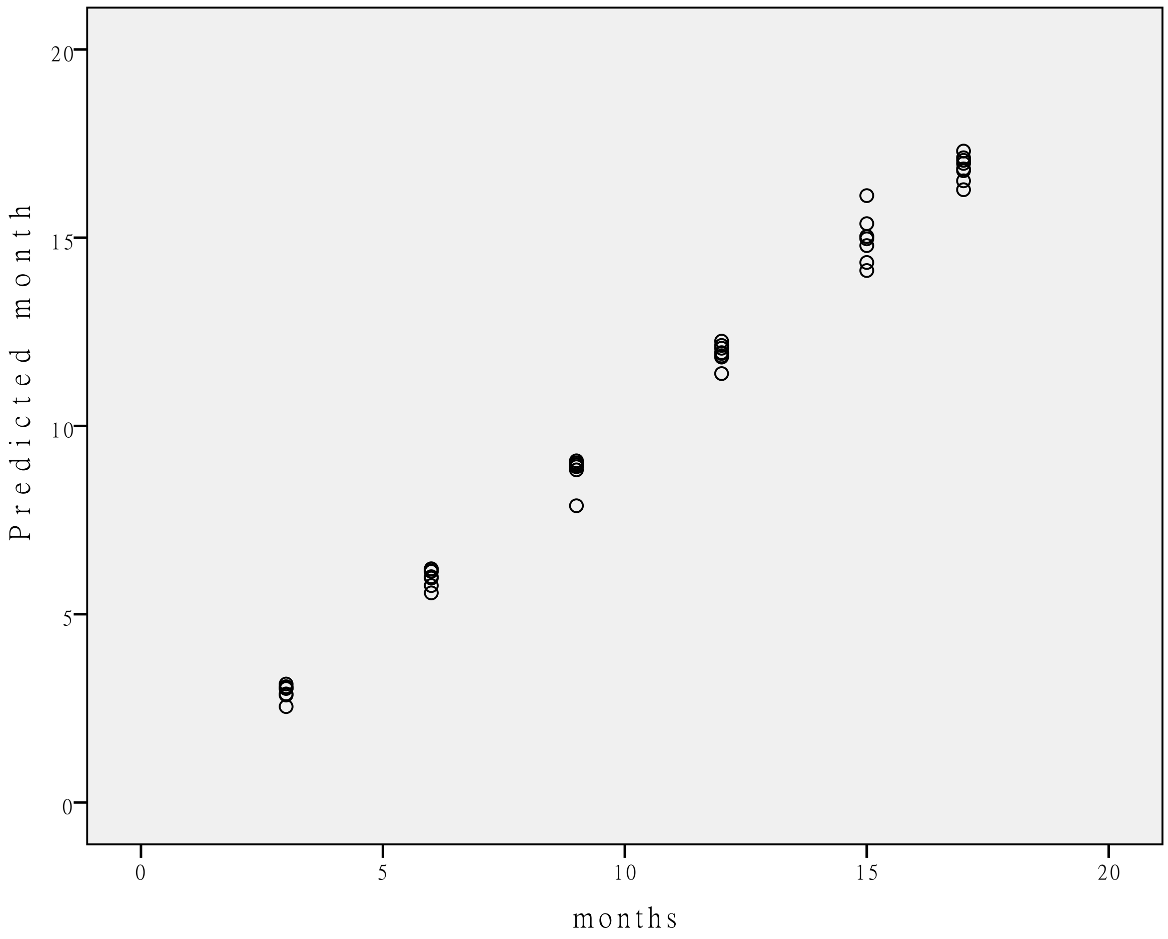

Predicted month
20
15
10
5
0
0
5
10
15
20
months

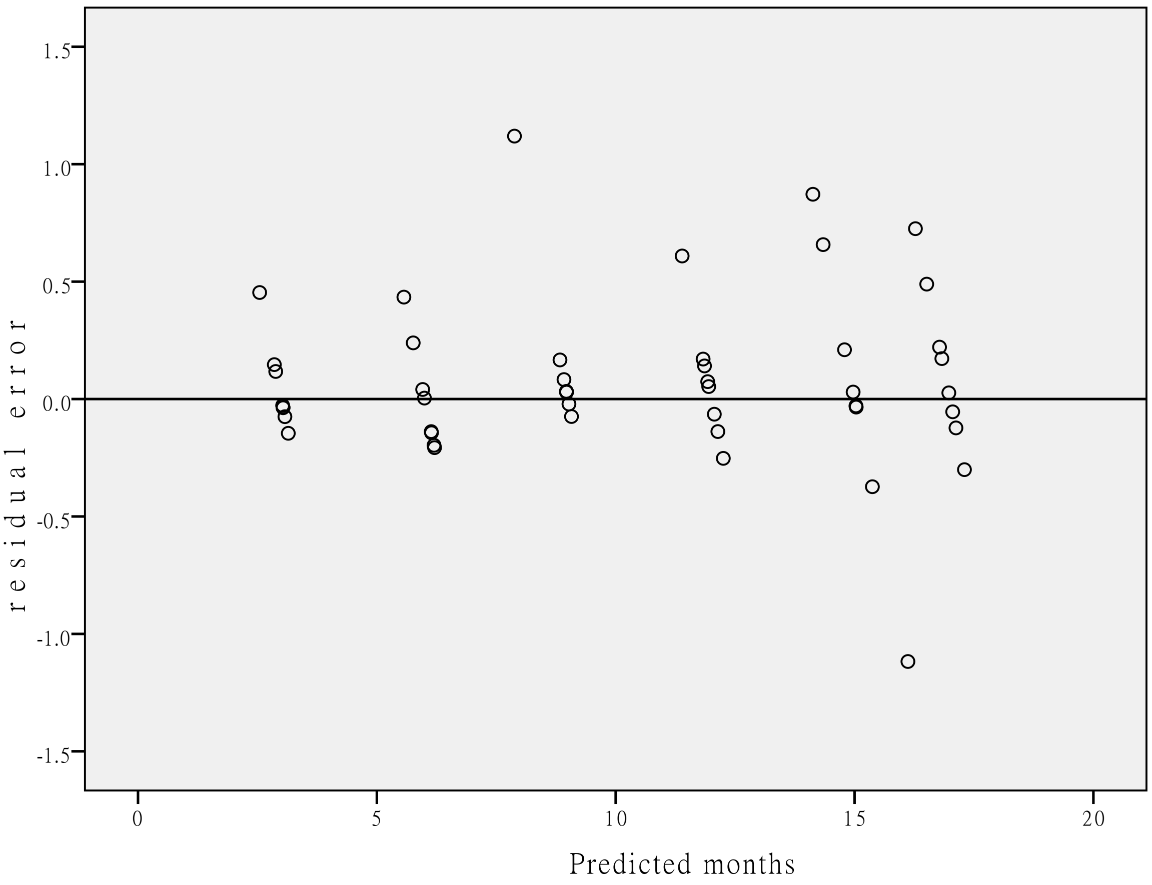

1.5
1.0
0.5
0.0
-0.5
-1.0
-1.5
residual error
0
5
10
15
20
Predicted months

自变量的重要性

	重要性	标准化的重要性
body weight	.012	18.8%
B M D	.021	33.3%
Ultimate deflection	.010	15.7%
Maximal load	.020	32.8%
Wet weight/volume	.020	32.0%
Dry weight/volume	.029	45.9%
Asht weight/volume	.024	38.7%
ash weight/dry weight	.016	26.4%
BV/TV	.028	44.9%
BS/BV	.034	54.0%
Tb.Th	.019	29.9%
Tb.N	.008	13.0%
Tb.Sp	.020	31.4%
Serum OPG	.014	21.9%
Serum RANKL	.026	40.9%
Serum OPG/RANKL	.015	24.3%
Serum TGF-β1	.020	31.8%
Serum bALP	.044	71.2%
Serum TRACP	.013	21.0%
Serum bALP/TRACP	.041	66.1%
ALP	.034	54.8%
TRACP	.035	55.8%
ALP/TRACP	.027	42.9%
RANKL protein	.016	25.7%
RANKL mRNA	.018	28.7%
OPG protein	.013	21.6%
OPG mRNA	.018	29.4%
OPG/RANKL protein	.023	36.8%
OPG/RANKL mRNA	.062	100.0%
IGF-1 protein	.022	35.9%
IGF-1 mRNA	.044	69.9%
M-CSF protein	.027	43.1%
M-CSF mRNA	.021	33.2%
TGF-β1 protein	.029	46.2%
TGF-β1 mRNA	.014	22.0%
BMP-2 protein	.015	23.9%
BMP-2 mRNA	.025	39.7%
Atp6v0d2 protein	.010	15.4%
Atp6v0d2 mRNA	.027	43.5%
Wnt5a protein	.059	94.5%
Wnt5a mRNA	.029	46.9%
